# Mature Mediastinal Teratoma Complicated with Spontaneous Rupture into the Pleural Cavity: A Report of a Pediatric Case

**DOI:** 10.5334/jbsr.3836

**Published:** 2025-02-25

**Authors:** Filipa Lima Coelho, Mário Rui Correia, Fátima Carvalho

**Affiliations:** 1 Clínica de Imagiologia Diagnóstica e de Intervenção, Centro Hospitalar Universitário de Santo António, Unidade Local de Saúde de Santo António, Porto, Portugal; 2 Serviço de Cirurgia Pediátrica, Centro Materno Infantil do Norte Albino Aroso, Centro Hospitalar Universitário de Santo António, Unidade Local de Saúde de Santo António, Porto, Portugal

**Keywords:** mediastinal teratoma, pleural effusion, spontaneous rupture

## Abstract

*Teaching point:* This is a review of the typical radiological features of a mature mediastinal teratoma with a rare presentation of spontaneous pleural rupture.

## Case History

**Figure 1:**
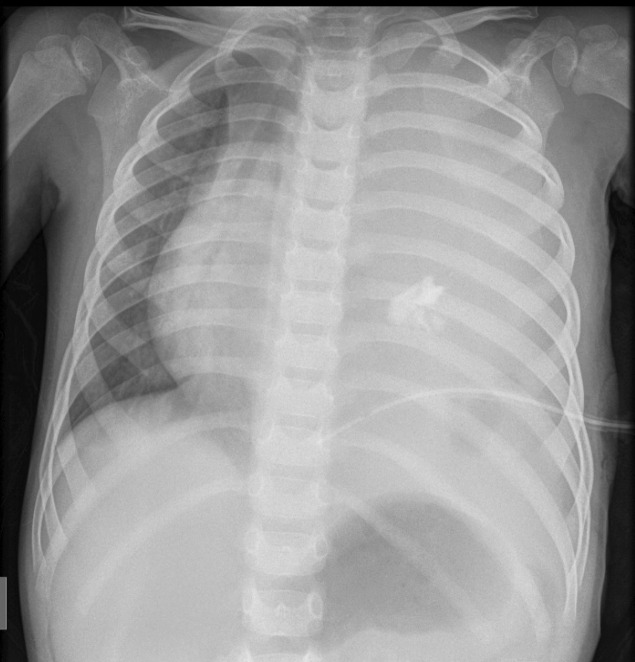
Preoperative CXR.

A 3‑year‑old boy presented with a sudden onset of cough and dyspnea. There was no history of fever, hemoptysis, chest pain, or trauma. Chest radiograph (CXR) showed an opaque left hemithorax, with a significant pleural effusion, loss of the left cardiac border silhouette, and shifting of the mediastinum to the right. Coarse calcifications were visible in the left hemithorax (Figure 1).

**Figure 2:**
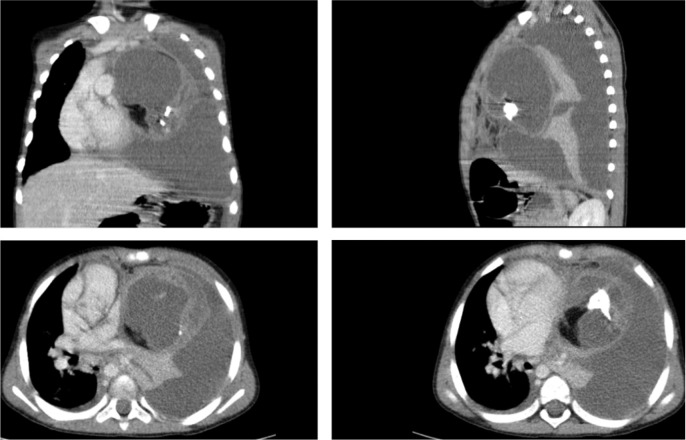
Multiplanar contrast‑enhanced CT scan.

Chest computed tomography (CT) revealed a heterogeneous anterior mediastinal cystic mass, with areas of calcification, soft tissue, and fat density. Also, left‑side pleural effusion and obliteration of the left main bronchus were noted, with left lung collapse (Figure 2).

The imaging features and acute onset of symptoms suggested the diagnosis of a mediastinal teratoma with spontaneous rupture into the pleural cavity.

During hospitalization, a left thoracentesis was performed, with the removal of 100 cc of yellowish fluid, and with clinical improvement. Cultures of the pleural fluid were sterile. Preoperative serum levels of alpha‑fetoprotein were normal.

A left thoracotomy was performed four months later, after pulmonary stabilization with conservative treatment. The anterior mediastinal mass was successfully dissected, measuring 10 cm in its largest diameter.

Pathologic examination showed a unilocular tumor, composed of a variety of tissues from all three germ cell layers, confirming the diagnosis of mature teratoma.

**Figure 3:**
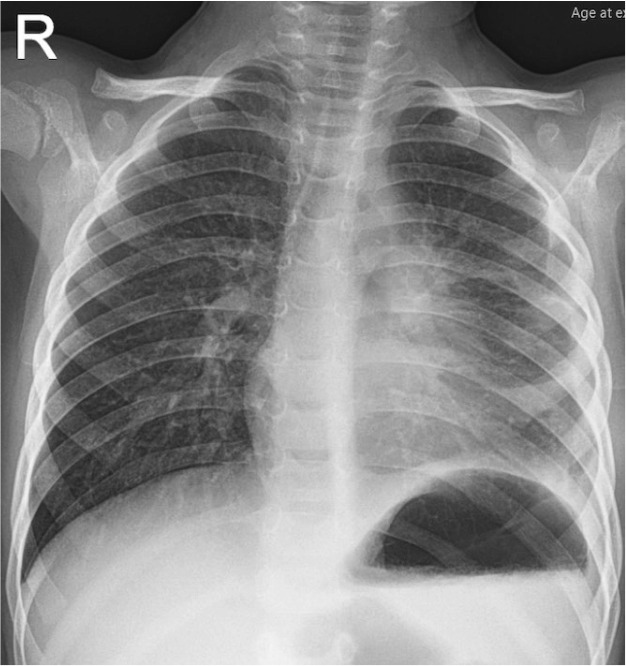
Postoperative CXR.

The postoperative CXR showed resolution of pleural effusion and mediastinal mass (Figure 3). The patient fully recovered.

## Comment

Mature teratoma is the mediastinum’s most frequent primary germ cell tumor, containing derivatives of all three germ layers [1].

Complications developing from these tumors are uncommon, and reports of spontaneous rupture of mediastinal teratomas into the adjacent structures, such as the pleural cavity, tracheal tree, pericardium, or lung parenchyma, among pediatric populations, are even rare [1].

The symptoms of a rupture teratoma include acute onset of severe chest pain, dyspnea, and hemoptysis. CXR shows typical findings of a mediastinal mass associated with unilateral pleural effusion [1].

The majority of unruptured mediastinal teratomas appear as a homogeneous encapsulated mass on CT, containing fat, calcifications, soft tissue, fluid, or any combination of these. In contrast, ruptured teratomas demonstrate heterogeneous densities of internal components. Other imaging findings of mediastinal teratoma rupture include pleural effusion, consolidation, or compressive atelectasis of the adjacent lung [1].

CT imaging largely contributes to detection and characterization, appropriate delineation of the mass to the adjacent tissue, such as the lung and pleural cavity, and to the surgical planning of this rare complication of mediastinal teratoma.

Magnetic resonance imaging (MRI) is a valuable alternative to CT for characterizing and delineation of teratomas since fat components are promptly identified as high signal intensity areas on T1‑weighted images that become suppressed with fat saturation.

Surgical management is the optimal treatment for ruptured mediastinal teratoma. Surgery may be complicated due to the leakage of the ruptured components into the thoracic cavity, causing adhesions in the adjacent anatomical structures [1].
